# Human biomonitoring of neonicotinoid exposures: case studies after the use of a spray-agent to ornamental plants and a topical medication to pets

**DOI:** 10.3389/fpubh.2023.1321138

**Published:** 2024-01-23

**Authors:** Sonja A. Wrobel, Stephan Koslitz, Daniel Bury, Heiko Hayen, Holger M. Koch, Thomas Brüning, Heiko U. Käfferlein

**Affiliations:** ^1^Institute for Prevention and Occupational Medicine of the German Social Accident Insurance, Ruhr University Bochum (IPA), Bochum, Germany; ^2^Institute of Inorganic and Analytical Chemistry, University of Münster, Münster, Germany

**Keywords:** risk assessment, daily intake, absorbed dose, insecticide, urine, LC–MS/MS, pet, ornamental plants

## Abstract

Acetamiprid (ACE) and imidacloprid (IMI) are insecticides of global importance and are used as spray and watering agents for ornamental plants to control biting and sucking insects or as topical medications on pets to remove and control fleas. Human biomonitoring data on ACE and IMI exposures when applying these products are limited. We investigated exposures to ACE and IMI in male volunteers after the domestic application of either an ACE-containing agent or an IMI-containing spot-on medication. Complete and consecutive urine samples were collected for up to 56 h after application. Urine samples were analyzed for ACE, IMI, and their respective metabolites (*N*-desmethyl-ACE, IMI-olefin, and sum of 4−/5-hydroxy-IMI) by liquid chromatography–tandem mass spectrometry. Fairly uniform concentrations of *N*-desmethyl-ACE could be observed before and after orchid treatment, so that an ACE exposure associated with orchid treatment can most likely be excluded. In contrast, after the application of the IMI-containing medication, elevated concentrations of IMI, 4−/5-hydroxy-IMI, and IMI-olefin were quantified in urine samples post-20 h with maximum concentrations of 3.1, 14.9, and 8.0 μg/g creatinine, respectively, well above general background levels. Nevertheless, the IMI intake (10.6 μg/kg bw), calculated from the excreted amounts, was around five times below the current European acceptable daily intake. Based on the case results here, household exposures to ACE and IMI after spray treatment of ornamental plants and anti-flea treatment of dogs can be regarded as low and safe. However, people regularly applying neonicotinoid-containing formulations, such as professional gardeners and employees in animal shelters, should be studied in more detail.

## 1 Introduction

The domestic use of insecticides is common, e.g., to control pests in ornamental plants and pets (1–3). Therefore, human exposure to insecticides is plausible and can occur in non-occupational settings as well. Nevertheless, actual information on exposure levels in humans after domestic use of insecticides is limited (2, 3). Additionally, there is continued discussion and rising concern about the environmental impact of veterinary medication (4, 5).

**Figure 1:**
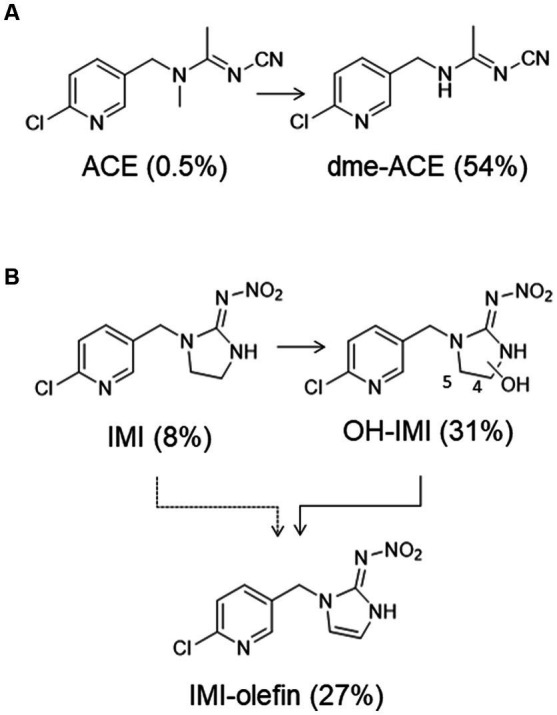
Human metabolism of (**A**) ACE and (**B**) IMI. Numbers in parentheses represent the urinary excretion fractions (F_ue_s) relative to an oral dose, as previously published (8). For IMI, the summed biomarkers excreted in urine account for 66% of the oral dose.

Acetamiprid (ACE) and imidacloprid (IMI) are two neonicotinoid insecticides (NNIs), which are often used as active ingredients in spray and watering agents for ornamental plants and veterinary medication to remove and control fleas in Germany (6, 7). In humans, prior to urinary excretion, ACE is mainly metabolized to *N*-desmethyl (dme)-ACE (Figure 1A) and IMI to 4−/and 5-hydroxy (OH)-IMI and IMI-olefin (Figure 1B) (8).

While human biomonitoring data on the exposure to NNIs of the general population became available in recent years (9–11), exposure data related to sources, especially domestic use of these insecticides, are limited. Therefore, the main aim of this research was to investigate if exposure to ACE and IMI occurs in two typical household scenarios, i.e., using an ACE-containing spray-treating agent on orchids or an IMI-containing spot-on medication on a dog. For this purpose, the urinary excretions of ACE and IMI, as well as their metabolites, were followed by a single male volunteer for each of the aforementioned applications.

## 2 Materials and method

### 2.1 Applied products

An ACE-containing spray-agent for ornamental plants (‘Substral Celaflor – Schädlingsfrei CAREO Konzentrat’, Evergreen Garden Care Deutschland GmbH, Mainz, Germany, Supplementary Figure 1) and an IMI-containing spot-on solution product for dogs (‘Advantix’, KVP Pharma + Veterinär Produkte GmbH, Kiel, Germany, Supplementary Figure 2) were purchased by the volunteers for the intended use at home (´over-the-counter´ products).

### 2.2 Study design

For applying a spray agent on ornamental plants (‘ACE case study’), 5 mL of the ACE-containing spray agent (5 g/L; 0.5wt.–%) were diluted in 500 mL of tap water (final concentration of the active ingredient: approximately 50 mg/L) according to the manufacturer’s instructions. Ornamental plants (orchids) were then sprayed directly on the leaves and roots from close range to treat for small infestations of leaf scale aphids and mealybugs (Supplementary Figure 3). Both spraying and watering of the ACE formulation were recommended by the manufacturer, depending on the crops and application site. Although watering was recommended for ornamental plants in pots indoors, we opted for spraying to possibly create a scenario with increased exposure. For topically applying the medication on pets (‘IMI case study’), the ready-to-use IMI-containing spot-on solution (2.5 mL containing 1 g/L of IMI) was applied at three spots to the dog’s back directly on the skin by manually splitting the hair according to the manufacturer’s instructions (Supplementary Figure 4). No gloves were worn in either case study (ACE or IMI application), as this was not explicitly recommended by the manufacturers.

Orchids were simply air-dried after product application and no direct contact occurred later on. The first dog contact in terms of petting and cuddling after treatment was reported at 8.5 h post-application.

Urine samples were collected directly before the application of the NNI-containing agents (*t*_0_) and consecutively and completely during the following 48 (ACE case study) or 56 h (IMI case study). The time periods were set to a minimum of 48 h to stay in line with our previously performed studies in volunteers after the oral dosage of neonicotinoids (8, 9). Urine samples were stored frozen (−20°C) in 250 mL polyethylene (PE) containers until analysis. The study was carried out according to the Code of Ethics of the World Medical Association (Declaration of Helsinki, written informed consent (IRB Reg. No.: 18-6680-BR)).

### 2.3 Urine analyses

Quantification of ACE, IMI, and their specific metabolites dme-ACE, OH-IMI, and IMI-olefin was performed by stable isotope dilution analysis using online-solid phase extraction (SPE)-LC–MS/MS as previously published (10). In brief, stable isotope-labeled internal standards, buffer, and pure β-glucuronidase from *E. coli* K12 were added to urine samples, and the samples were then incubated in a water bath at 37°C for 1 h for the hydrolysis of glucuronic acid conjugates. After incubation, samples were frozen overnight, thawed, equilibrated to room temperature, and centrifuged (1900 *g*, 10 min). A measure of 50 μL of the supernatant was injected into the LC–MS system. Limits of quantification (LOQ) were 0.06 μg/L (ACE), 0.15 μg/L (dme-ACE), 0.19 μg/L (IMI), 1.00 μg/L (OH-IMI), and 2.10 μg/L (IMI-olefin). The creatinine concentration of the urine samples was determined by the Jaffé method (L.u.P. GmbH Labor und Praxisservice, Bochum, Germany).

### 2.4 Estimation of NNI intakes

To back-calculate the NNI intakes (in μg/kg body weight) from urinary biomarker excretion, previously published quantitative toxicokinetic data on ACE and IMI derived in humans, including urinary excretion fractions (F_ue_s), were used (8). NNI intakes were calculated over the complete study time (up to 48 or 56 h after application) using Equation 1.

(1) $$NNI\;intake=\frac{\sum \left(c_{i-ne}\times V_{i}\;\right)}{F_{ue-ne}\times bw}\times M_{n}$$

with *c_i-ne_* being either the dme-ACE concentration or sum of the excreted IMI biomarker concentrations at time point i in mol/L, *V_i_* is the volume of the corresponding urine sample in L at time point *i*, F_ue-ne_ is the urinary excretion fractions of dme-ACE or the sum of the individual F_ue_s of the IMI biomarkers excreted via urine within 48 h after oral application relative to the incorporated NNI dose (see Figure 1), bw is the body weight of the volunteer in kg (see Supplementary Table 1), and M_n_ is the molar masses of either ACE or IMI. Molar masses of ACE, dme-ACE, IMI, OH-IMI, and IMI-olefin were 223, 209, 256, 272, and 254 g/mol, respectively.

## 3 Results

### 3.1 ACE case study

**Figure 2:**
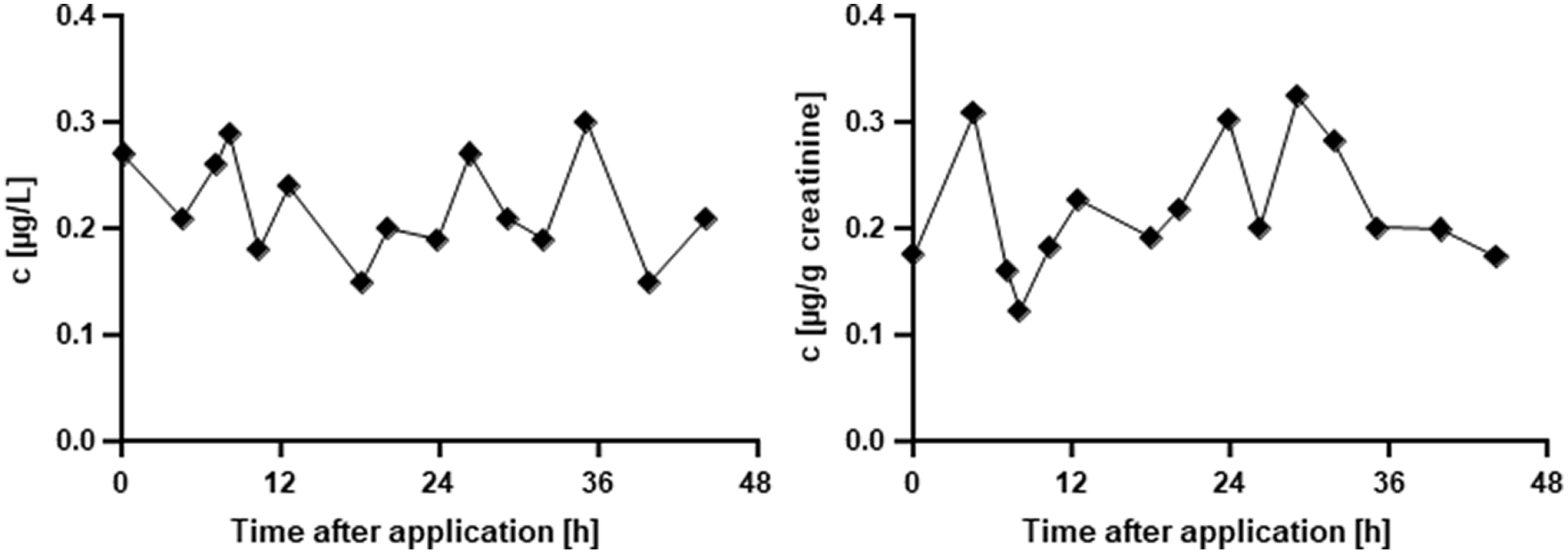
Urinary concentration of dme-ACE after in-house use of an ACE-containing spray agent; left, absolute concentrations in μg/L; right, creatinine adjusted concentrations in μg/g creatinine. Values below LOQ are not shown.

In the ACE case study, ACE itself was not quantifiable above the LOQ in any of the samples. In contrast, its metabolite dme-ACE was already quantifiable in the *t*_0_ sample and continuously until 44 h after spraying (Figure 2). In the last two samples, dme-ACE was below the LOQ. The maximum measured concentration (*c*_max_) was 0.32 μg/g creatinine.

Fairly uniform concentrations of dme-ACE were observed over the whole study period without an identifiable excretion pattern for both volume- and creatinine-adjusted concentrations. The total excretion of dme-ACE over the observation time was 0.80 μg, which corresponded to an ACE intake of 0.01 μg/kg body weight.

### 3.2 IMI case study

**Figure 3:**
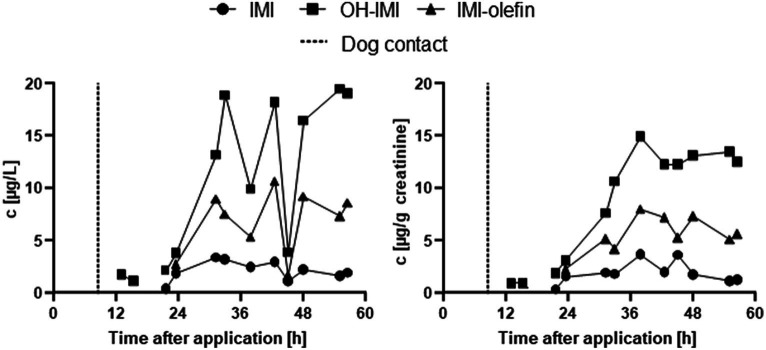
Urinary excretion of IMI, OH-IMI, and IMI-olefin after the application of an IMI-containing spot-on solution on a dog; left, absolute concentrations in μg/L; right, creatinine adjusted concentrations in μg/g creatinine. The dashed line represents the time point (8.5 h) indicating the first skin contact with the dog after treatment. Values below LOQ are not shown.

In the IMI case study, the concentrations of IMI, OH-IMI, and IMI-olefin were below the LOQ at *t*_0_. OH-IMI was the first metabolite to emerge with concentrations above the LOQ in two samples at 13 and 15 h after dog treatment (or 4.5 and 6.5 h after petting and cuddling with the dog). All three analytes were then quantifiable in all urine samples 20 h post-application (or 11.5 h after the first post-application contact with the dog); see Figure 3.

A clear treatment-associated time-concentration curve was observed for all three urinary IMI exposure biomarkers starting 20 h after the application (or 11.5 h after the first post-application contact with the dog). Creatinine adjustment (for differing urinary dilutions) leads to a considerable smoothing of the curve compared to unadjusted μg/L levels. Creatinine-corrected urinary concentrations rather constantly increased to reach their maximum 38 h after the application, with corresponding *c*_max_ of 3.1, 14.9, and 8.0 μg/g creatinine for IMI, OH-IMI, and IMI-olefin, respectively. Thereafter, the levels slowly decreased but remained well above the LOQ 56 h after application. The total excretion of IMI, OH-IMI, and IMI-olefin (until the last sampling point 56 h post-application) was 73.5, 439.6, and 212.0 μg, respectively, corresponding to an oral dose equivalent IMI intake of 10.6 μg/kg body weight based on the summed three urinary biomarkers.

### 3.3 Discussion

In the ACE case study, no unchanged ACE was found in any of the urine samples at concentrations above the LOQ (0.06 μg/L). This is not surprising, as ACE is known to be rapidly metabolized into dme-ACE (8, 12), and the F_ue_ of the unchanged ACE is very low (0.5%, see Figure 1). Contrary to that, dme-ACE was found in almost all urine samples, including the one at *t*_0_ before the spray application (Figure 2), thus suggesting background exposures to ACE (possibly via diet) in the volunteer and already prior to spraying the orchids. All measured concentrations were rather close to the LOQ of 0.15 μg/L or even below the LOQ (*n* = 3), which also explains the absence of unchanged ACE. All dme-ACE concentrations above the LOQ (*n* = 13) (median 0.21 μg/L, 95th percentile 0.29 μg/L) were well in the background range of ACE exposures previously reported in individuals from the German general population (median 0.38 μg/L, 95th percentile of 0.83 μg/L) (10). Overall, the lack of a classical excretion pattern and the rather uniform concentrations of dme-ACE suggest constant environmental ACE exposures in our volunteers that were not related to the spray-treating of the orchids but, most likely, to diet. The estimated ACE intake for our volunteer based on the excretion of dme-ACE (0.01 μg/kg body weight) was comparable to the intakes previously estimated for the German general population (median DI: 0.03 μg/kg body weight/day) (8) and thus well below the current acceptable daily intakes of the European Union of 25 μg/kg body weight/day for ACE (13).

In the IMI case study, we observed classical post-exposure excretion patterns in terms of, first, increasing concentrations followed by decreasing levels for all analytes (Figure 3). Neither IMI nor its metabolites, OH-IMI and IMI-olefin, were quantifiable in the urine sample before the dog was treated (*t*_0_). This result is in line with previous findings in Germany, where most investigated urine samples did not show any IMI exposure biomarkers. In contrast to ACE, the use of IMI has been restricted to greenhouse uses in the European Union since 2013 (14) due to its toxicity in pollinators, thus limiting the presence of IMI in crops and, consequently, background exposures of the general population of Germany/Europe via diet. The excretion pattern of IMI and its metabolites after topical application of the spot-on solution to a dog is therefore clearly associated with the aforementioned treatment. Interestingly, IMI biomarkers started to be detected in urine only after petting the dog at 8.5 h post-treatment rather than directly after the topical application. We therefore assume that the petting of the treated dog is the cause of exposure rather than the original application of the spot-on agent (Figure 3). The IMI intake was calculated based on the sum of the urinary IMI, OH-IMI, and IMI-olefin in urine and their known urinary excretion fraction (15). However, as visible from the excretion kinetics (Figure 3), the urinary excretion of IMI has not been completed within the sample collection period. There are several reasons for this: From our oral dosing study, we know that the elimination half-times of IMI and its metabolites after oral dosage are rather long (12–23 h). Given the delayed uptake in our study, a total collection time of 56 h might not have been sufficient. Dermal uptake must be considered the major route of exposure in our study, similar to other studies that previously investigated exposures to active compounds in flea-controlling veterinary products (3). Compared to oral uptake, dermal uptake is slower and results in a delayed urinary excretion of IMI and its metabolites. However, human toxicokinetics after the dermal uptake of IMI have not yet been investigated in detail. Moreover, cuddling with dogs occurs infrequently and therefore cannot be considered a single exposure event (such as the spot application itself or the “first” cuddling of the dog). Because several succeeding exposure events occur at infrequent intervals, we must assume that more than 10.6 μg/kg body weight of IMI will be taken up (although the total uptake is distributed across several days post-application). Nevertheless, in our single treatment study, we could evidence the uptake of IMI in a dog owner after topically applying an ´over-the-counter´ product for controlling fleas. Overall, this single treatment did not result in an exceedance and was about a factor of 5 below the current acceptable daily intake of the European Union of 60 μg/kg body weight/day for IMI (13).

All data presented here are based on a single volunteer for each substance only and should be regarded qualitatively. Further studies including more volunteers and under varying exposure situations would be needed to assess the range of exposure quantitatively and the toxicological significance of these exposures.

## 4 Conclusion

Human biomonitoring has the advantage of reliably quantifying the total body burden that can occur during the use of NNI products in occupational or private environments, irrespective of the complexity of potential exposure routes (dermal, oral, and inhalation), capturing all routes and sources of exposure. Our data give first insights into ACE and IMI exposures after two different, even though specific household applications. The applications were carried out in such a way that was more likely to result in increased exposure, i.e., preparing and applying all solutions without dermal protection (no use of gloves) and, in the case of the ACE case study, spraying the plants rather than watering them. For the use of the ACE-containing plant protection product, we found no additional treatment-related exposure on top of the general background exposure to ACE. For the dog treatment with IMI, we clearly found exposures were almost exclusively related to the cuddling of the dog rather than the direct topical application of the flea-control product itself. Furthermore, we have to assume that multiple exposure routes (inhalation of dog dander and/or fine hair and dermal penetration) contributed to the total exposure, which would have been difficult to capture with exposure assessment techniques other than human biomonitoring. Therefore, although these two case studies certainly cannot be generalized with regard to every single domestic exposure situation, our study reveals that the use of spot-on medications must be considered more relevant than spray-treating ornamental plants indoors. However, the back-calculation of oral intake equivalents by reverse dosimetry from urinary biomarkers did not indicate critical IMI intake levels yet for the studied dog owner after a single application.

Generally, future studies should investigate the dermal absorption of neonicotinoids. In addition, settings presumably associated with increased exposures, such as occupational exposures of farmers, gardeners, and employees in animal shelters and veterinary practices, should be studied in more detail, and a higher number of study subjects should be used as close and continued contact with treated pets or contact with multiple treated pets might lead to cumulated exposures approaching or exceeding the ADI for IMI.

## Data availability statement

The raw data supporting the conclusions of this article will be made available by the authors, without undue reservation.

## Ethics statement

The studies involving humans were approved by Ethik-Kommission der Medizinischen Fakultät der Ruhr-Universität Bochum. The studies were conducted in accordance with the local legislation and institutional requirements. The participants provided their written informed consent to participate in this study.

## Author contributions

SW: Conceptualization, Data curation, Formal analysis, Investigation, Methodology, Visualization, Writing – original draft, Writing – review & editing. SK: Conceptualization, Visualization, Writing – review & editing. DB: Supervision, Visualization, Writing – review & editing. HH: Supervision, Writing – review & editing. HKo: Resources, Supervision, Writing – review & editing. TB: Resources, Writing – review & editing. HKä: Project administration, Resources, Supervision, Visualization, Writing – review & editing.
