# Supplementary material for: Human biomonitoring of neonicotinoid exposures: case studies after the use of a spray-agent to ornamental plants and a topical medication to pets
Source: Front Public Health. 2024 Jan 23;11:1321138. doi: 10.3389/fpubh.2023.1321138 (PMC10844535; doi:10.3389/fpubh.2023.1321138)
Supplement: Supplementary file 1 [file Data_Sheet_1.PDF]

Supplementary Material to

Human biomonitoring of neonicotinoid exposures: case studies after use of a spray-agent on ornamental plants and a topical medication on pets

Sonja A. Wrobel[1], Stephan Koslitz[1], Daniel Bury[1], Heiko Hayen[2], Holger M. Koch[1], Thomas Brüning[1], Heiko U. Käfferlein[1,*]

[1]Institute for Prevention and Occupational Medicine of the German Social Accident Insurance, Ruhr University Bochum (IPA), Bürkle-de-la-Camp-Platz 1, 44789 Bochum, Germany

[2]Institute of Inorganic and Analytical Chemistry, University of Münster, Corrensstraße 48, 48149 Münster, Germany

*** Correspondence:**
Heiko U. Käfferlein
heiko.kaefferlein@dguv.de

1 Supplementary Data

1.1 Product information of the used NNI-containing agents

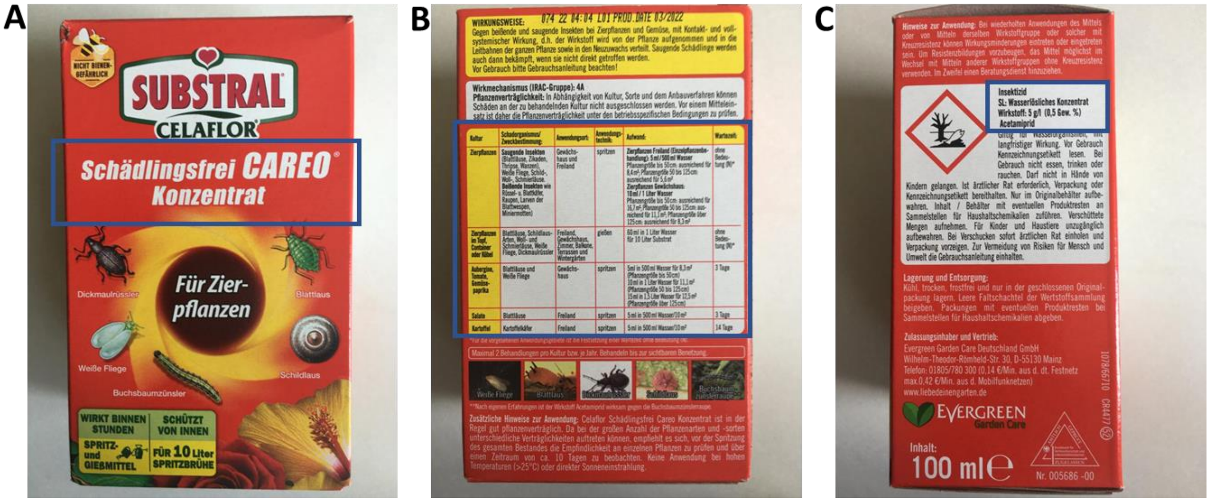

Supplementary Figure 1. Pictures of the commercially purchased spray-agent containing ACE for use on ornamental plants. Blue boxes indicate relevant information on the front (**A)**, back (**B**), and side (**C**) of the package indicating the active ingredient (‘Acetamiprid; 5 g/L; 0.5wt.-%’) and its recommended use.

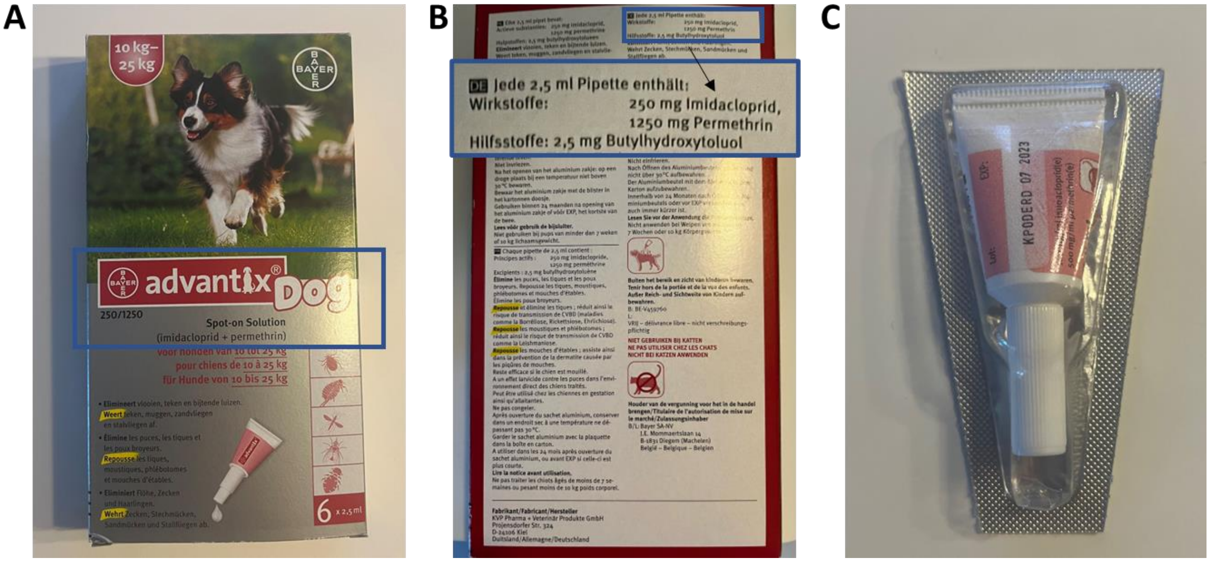

Supplementary Figure 2. Pictures of the commercially purchased spot-on solution containing IMI for use on dogs. Blue boxes indicate relevant information. **A**: Front of the package showing the name of the veterinary product (‘Advantix - Dog’). **B**: Back of the package listing the ingredients of the solution (2.5 mL solution contains 250 mg of IMI). **C**: Pipette containing the spot-on solution.

1.2 Photo series on the application of solutions containing NNIs

Preparation

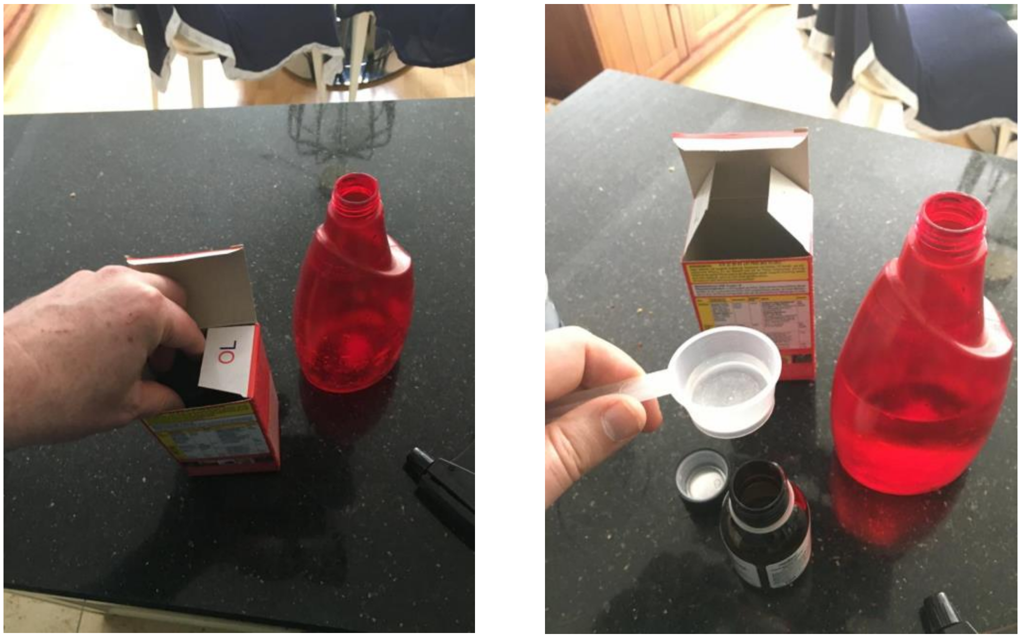

Treatment

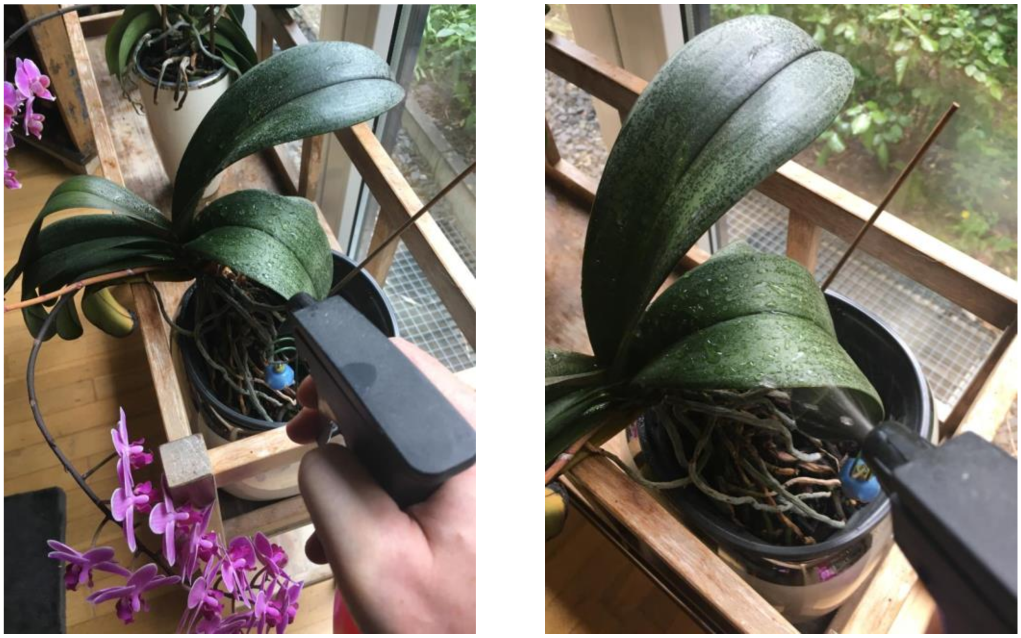

Supplementary Figure 3. Depiction of the plant treatment using an ACE-containing spray-agent. Preparation of treatment solution (top) and spray-treating orchids (bottom). Due to aerial roots and to avoid stagnant water, orchids, in deviation from the manufacturer's specifications for indoor ornamental flowers, were sprayed rather than watered.

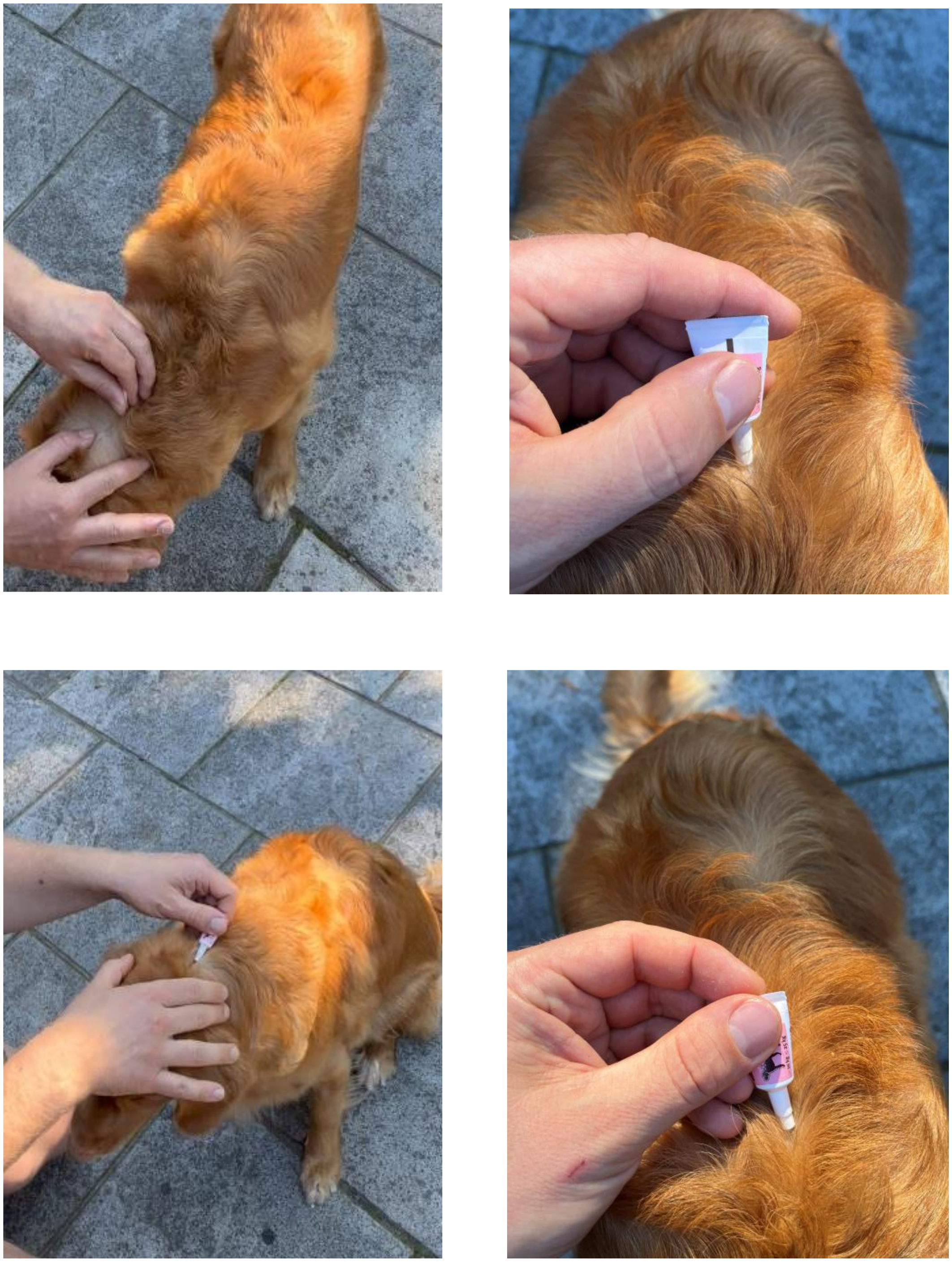

Supplementary Figure 3. Depiction of the dog treatment using an IMI-containing spot-on solution.

1.3 Volunteer and sample information

	Age [years]	Body weight [kg]	No. of samples	Total volume [L]
ACE application	52	83	16	4.943
IMI application	40	100	16	5.055

Supplementary Table 1. Age and body weight of the volunteers at time of the application and indication of the number of samples and the total urine volume during sampling.
